# Association of the Risk of Primary Sjögren's Syndrome With Fibrocystic Breast Disease: A Nationwide, Population-Based Study

**DOI:** 10.3389/fmed.2021.704593

**Published:** 2021-07-01

**Authors:** Hsin-Hua Chen, Hsian-Min Chen, Ching-Heng Lin, Kuo-Tung Tang, Der-Yuan Chen, James Cheng-Chung Wei, Wen-Cheng Chao

**Affiliations:** ^1^Department of Medical Research, Taichung Veterans General Hospital, Taichung, Taiwan; ^2^Division of Allergy, Immunology and Rheumatology, Department of Internal Medicine, Taichung Veterans General Hospital, Taichung, Taiwan; ^3^Institute of Biomedical Science and Rong Hsing Research Centre for Translational Medicine, Chung Hsing University, Taichung, Taiwan; ^4^Department of Industrial Engineering and Enterprise Information, Tunghai University, Taichung, Taiwan; ^5^Big Data Center, Chung Hsing University, Taichung, Taiwan; ^6^Department of Medical Research, Center for Quantitative Imaging in Medicine, Taichung Veterans General Hospital, Taichung, Taiwan; ^7^Department of Computer Science and Information Engineering, National United University, Miaoli, Taiwan; ^8^Department of Healthcare Management, National Taipei University of Nursing and Health Sciences, Taipei, Taiwan; ^9^Department of Public Health, College of Medicine, Fu Jen Catholic University, New Taipei City, Taiwan; ^10^Faculty of Medicine, National Yang-Ming University, Taipei, Taiwan; ^11^Rheumatology and Immunology Center, China Medical University Hospital, Taichung, Taiwan; ^12^School of Medicine, China Medical University, Taichung, Taiwan; ^13^Translational Medicine Laboratory, Rheumatology and Immunology Center, China Medical University Hospital, Taichung, Taiwan; ^14^Division of Allergy, Immunology and Rheumatology, Chung Shan Medical University Hospital, Taichung, Taiwan; ^15^Institute of Medicine, Chung Shan Medical University, Taichung, Taiwan; ^16^Institute of Integrative Medicine, China Medical University, Taichung, Taiwan; ^17^Department of Critical Care Medicine, Taichung Veterans General Hospital, Taichung, Taiwan; ^18^Department of Computer Science, Tunghai University, Taichung, Taiwan; ^19^Department of Automatic Control Engineering, College of Information and Electrical Engineering, Feng Chia University, Taichung, Taiwan

**Keywords:** Sjögren's syndrome, mammary gland, mastitis, fibrocystic breast disease, epidemiology

## Abstract

**Objective:** Primary Sjögren's syndrome (pSS) is characterized by exocrine glandular inflammation; however, the association between preceding mammary-gland-inflammation-related diseases and newly diagnosed pSS remains unexplored.

**Methods:** We used the 2003–2013 data retrieved from Taiwan's National Health Insurance Research Database (NHIRD) to conduct the present population-based study. We identified newly diagnosed pSS female patients during the 2001–2013 period, as well as age-matched (1:20) and propensity-score-matched (1:2) non-SS individuals (as controls). We explored the associations between pSS and a history of mastitis and fibrocystic breast disease by determining adjusted odds ratios (aORs) with 95% confidence intervals (CIs) using a conditional logistical regression analysis after controlling for potential confounders.

**Results:** We identified 9,665 patients with pSS and 193,300 age-matched non-SS controls, as well as 9,155 SS cases and 18,310 propensity-score-matched non-SS controls. We found that fibrocystic breast disease (aOR, 1.75; 95% CI, 1.63–1.88) were independently associated with incident SS, whereas mastitis and childbirth-associated breast infections were not associated with incident SS. We also found positive associations between SS and previously reported SS-associated diseases, including cardiovascular diseases, thyroid diseases, pancreatitis, bronchiectasis, infectious diseases, osteoporosis, and ankylosing spondylitis. In the propensity-score-matched populations, the associations between pSS and fibrocystic breast disease (aOR, 1.74; 95% CI, 1.58–1.91) remained consistent.

**Conclusion:** The present population-based study revealed a previously unexplored association between pSS and history of fibrocystic breast disease, and the finding highlights the need to survey pSS in patients with mammary-gland-inflammation-associated diseases.

## Background

Primary Sjögren's syndrome (pSS) is a highly prevalent autoimmune disease that affects approximately 1% of the general population and is characterized by chronic inflammation of exocrine glands, mainly of the salivary, lacrimal, and mammary glands (1–4). Unlike numerous studies that have explored xerophthalmia and xerostomia in relation to pSS, the association between the inflammatory disease of mammary gland and pSS remains unclear (5, 6).

Fibrocystic disease and mastitis are two leading benign breast diseases, which may result from infection, autoimmune diseases, and breast cancer (7–9). Fibrocystic breast change is a highly prevalent benign breast disease in both pre-menopausal and post-menopausal women; moreover, studies have found that fibrocystic breast change may lead to moderate-to-severe breast pain in nearly 10% of healthy women (7, 10). With regard to mastitis, lactational mastitis appears to be an infectious disease among women of a young age, whereas non-lactational mastitis tends to be found in women older than 40 years and may be associated with autoimmune disease (6, 11). Given that the majority of patients with pSS are older than 40 years at the time of diagnosis, there is a crucial need to address the association between the benign breast disease and the development of pSS. In the present population-based study, we used not only age-matched, but also propensity-score-matched study populations to investigate the association between the benign breast disease and pSS.

## Materials and Methods

### Ethics Statement

The Institutional Review Board of Taichung Veterans General Hospital in Taiwan approved the present study (approval number: CE19038A). The requirement for informed consent was waived because the used data were anonymised.

### Data Sources

The data used in this study were derived from the National Health Insurance Research Database (NHIRD), which contains reimbursement claims data from the National Health Insurance, a compulsory insurance with nationwide coverage that was established in 1997. The diagnoses of inpatients and outpatients were in accordance with the International Classification of Diseases, Ninth Revision, Clinical Modification (ICD-9-CM). Additionally, patients with major illnesses, including cancer and several autoimmune diseases including SS were issued a certificate of catastrophic illness after the examination of the required data by at least two rheumatologists, and co-payment was waived among patients with the aforementioned certificate in Taiwan. The data regarding catastrophic illness certificates, namely the Registry for Catastrophic Illness Patient Database (RCIPD), were stored within NHIRD. Moreover, the NHIRD constructed a representative database of 1 million individuals who were randomly selected among insured residents who received medical services in 2000 (Longitudinal Health Insurance Database, LHID 2000). In the present study, we used RCIPD to identify patients with SS, and LHID 2000 to select matched non-SS controls. The index date was the date of the first pSS diagnosis among pSS cases and the date of the first ambulatory visit in the index year among non-SS controls.

### Study Design and Participants

**Figure 1:**
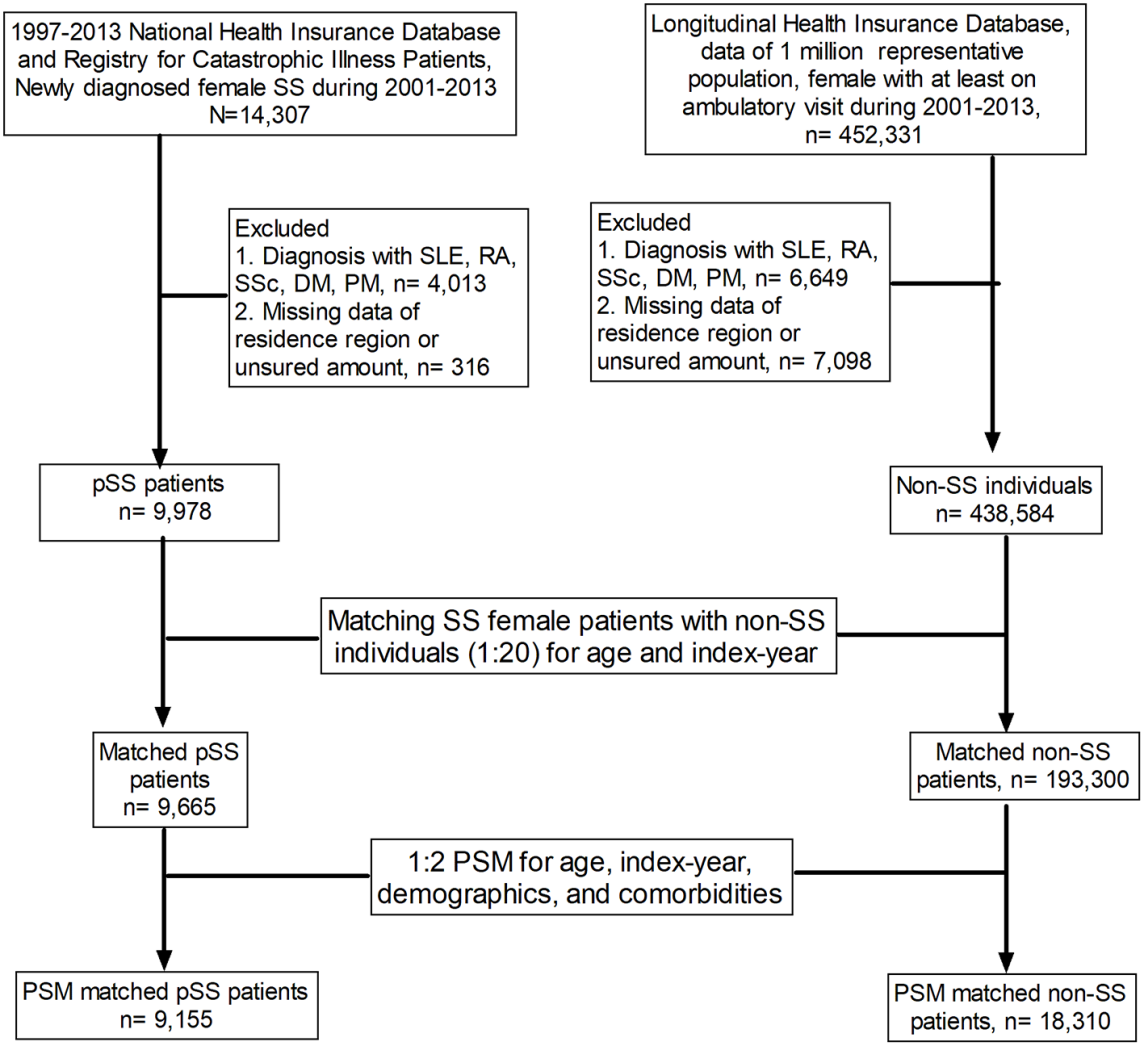
Flow chart of the study design. SS, Sjögren's syndrome; SLE, systemic lupus erythematosus; RA, rheumatoid arthritis; SSc, scleroderma; DM, dermatomyositis; PM, polymyositis; PSM, propensity score matching.

This case–control study was designed to investigate factors associated with pSS, as illustrated in the flow chart presented in Figure 1. We included all female patients diagnosed with pSS (*n* = 14,307; ICD-9-CM code 710.2) in Taiwan between 2001 and 2013 and excluded those with other autoimmune diseases, including systemic lupus erythematous, rheumatoid arthritis, scleroderma, dermatomyositis, and polymyositis, or missed detailed data regarding region of residence or insured amount; a total of 9,978 patients with pSS were identified as pSS cases. We used age matching and propensity score matching (PSM) in this study. We matched pSS cases and non-SS controls at a ratio of 1:20 for age and year of the index date. After matching, 9,665 pSS cases and 193,300 controls remained. We further attempted to reduce the impact of bias and confounders on the incidence of pSS through PSM, which was conducted at a ratio of 1:2 for age, index year, and selected comorbidities. Among the propensity-score-matched subjects, we identified 9,155 patients with pSS and 18,310 individuals without SS (Figure 1).

### Covariates

The covariates included in the adjustment in the regression model were age, common medical disorders, and benign breast diseases, including fibrocystic breast disease (ICD-9 code, 610.0-3), mastitis (ICD-9 code, 611.0), and childbirth-associated breast infection (ICD-9 code, 675). The presence of common medical disorder was defined as having one inpatient visit or at least three ambulatory visits with a corresponding ICD-9 code within 1 year prior to the index date. The adjusted known pSS-associated comorbidities consisted of cardiovascular diseases, thyroid diseases, bronchiectasis, pancreatitis, hepatitis C virus infection, osteoporosis, and ankylosing spondylitis. As previous studies have suggested a relationship between non-tuberculous infection and *Helicobacter pylori* infection and SS, we included these two diseases as covariates (12, 13). We also adjusted the analysis for socioeconomic status, including urbanization levels and insured income. Urbanization levels were categorized into three grades based on population density (people/km^2^), population ratio of elder subjects aged over 65 years, population ratio of subjects with college or higher educational levels, population ratio of agriculture workers and the number of physicians per 100,000 subjects (14). Importantly, we also used the frequency of outpatient visits 1 year prior to the diagnosis of pSS to adjust the potential surveillance bias (15).

### Statistical Analysis

The demographic data are presented as the mean ± standard deviation for continuous variables and as a percentage of patients for categorical variables. The differences were analyzed using Student's *t*-test for continuous variables and Pearson's χ^2^-test for categorical variables. The history of mammary-gland-inflammation-related diseases, as well as other potential pSS-associated diseases, in pSS cases compared with non-SS controls is presented as an adjusted odds ratio (aOR), as assessed using a conditional logistical regression analysis. All data were analyzed using the SAS software, version 9.3 (SAS Institute, Inc., Cary, NC, USA). Significance was set at *P* < 0.05.

### Sensitivity and Subgroup Analyses

The sensitivity analyses were conducted using distinct definitions for fibrocystic breast disease and mastitis. The significance of modification effect by the covariate was investigated by estimating the *p*-value of the coefficient associated with the product of each indicator of the covariate and the indicator of fibrocystic breast disease/mastitis using the Wald test.

## Results

### Characteristics of the Study Population

In the age- and index-year-matched population compared with non-SS controls, we found that SS cases had a similar distribution of urbanization levels and a low probability to have a lower insured amount (47.5 vs. 50.2%, *P* < 0.01), and were more likely to have mastitis (2.7 vs. 1.8%, *P* < 0.01) and fibrocystic breast disease (10.5 vs. 5.0%, *P* < 0.01), but not childbirth-associated breast infection (0.1 vs. 0.1%, *P* = 0.94). Regarding comorbidities and SS-relevant diseases, patients with SS were more likely to have cardiovascular diseases (type I DM: 0.2 vs. 0.1%, *P* = 0.03; coronary artery disease: 9.7 vs. 4.2, *P* < 0.01; cerebral vascular accident: 4.9 vs. 2.7%, *P* < 0.01; hyperlipidaemia: 16.0 vs. 7.3%, *P* < 0.01), thyroid diseases (hyperthyroidism: 3.9 vs. 0.7%, *P* < 0.01; thyroiditis: 1.3 vs. 0.1%, *P* < 0.01), pancreatitis (0.5 vs. 0.1%, *P* < 0.01), bronchiectasis (4.5 vs. 1.7%, *P* < 0.01), infectious diseases (hepatitis C virus infection: 2.3 vs. 0.6%, *P* < 0.01; non-tuberculous mycobacterial infection: 0.13 vs. 0.05%, *P* < 0.01; *H. pylori* infection: 1.6 vs. 0.6%, *P* < 0.01), osteoporosis (8.5 vs. 2.3%, *P* < 0.01), and ankylosing spondylitis (1.2 vs. 0.1%, *P* < 0.01) (Table 1).

**Table 1:** Baseline characteristics in the patients with primary SS and in the non-SS controls.

	**Before PSM**			**1:2 PSM**		
	**(1:20, age matching)**					
	**Non-SS**	**SS**	***P*-value**	**Non-SS**	**SS**	***P*-value**
	***n* =193,300**	***n* = 9,665**		***n* = 18,310**	***n* = 9,155**	
**Age**	53.3 ± 14.0	53.3 ± 14.0	1.00	53.5 ± 14.0	53.4 ± 14.0	0.74
**Urbanization**			0.21			0.69
Urban	60,105 (31.1)	2,925 (30.3)		5,587 (30.5)	2,784 (30.4)	
Suburban	90,096 (46.6)	4,545 (47.0)		8,514 (46.5)	4,302 (47.0)	
Rural	43,099 (22.3)	2,195 (22.7)		4,209 (23.0)	2,069 (22.6)	
**Low income**	96,977 (50.2)	4,590 (47.5)	<0.01	8,874 (48.5)	4,376 (47.8)	0.30
**Benign breast diseases**						
Fibrocystic disease	9,741 (5.0)	1,014 (10.5)	<0.01	1,049 (5.7)	936 (10.2)	<0.01
Mastitis	3,433 (1.8)	261 (2.7)	<0.01	361 (2.0)	240 (2.6)	<0.01
Breast infections associated with childbirth	286 (0.1)	14 (0.1)	0.94	17 (0.1)	12 (0.1)	0.36
**Interval between benign breast disease and SS (years)**						
Mastitis	5.3 ± 3.5	5.0 ± 3.4	0.09	5.5 ± 3.6	5.1 ± 3.4	0.22
Fibrocystic disease	5.7 ± 3.6	5.6 ± 3.7	0.56	5.5 ± 3.6	5.7 ± 3.7	0.20
Breast infections associated with childbirth	5.1 ± 3.7	5.2 ± 2.8	0.94	5.3 ± 3.7	4.8 ± 2.8	0.65
**Outpatient department visit^a^**	17.3 ± 13.3	25.6 ± 19.9	<0.01	20.9 ± 15.1	25.2 ± 19.8	<0.01
**Common medical disorders**						
Diabetes mellitus, type I	210 (0.1)	18 (0.2)	0.03	18 (0.1)	15 (0.2)	0.14
Diabetes mellitus, type II	16,703 (8.6)	853 (8.8)	0.53	1,570 (8.6)	790 (8.6)	0.88
Coronary artery disease	8,127 (4.2)	935 (9.7)	<0.01	1,545 (8.4)	829 (9.1)	0.09
Cerebral vascular accident	5,164 (2.7)	478 (4.9)	<0.01	867 (4.7)	438 (4.8)	0.86
Hyperlipidaemia	14,022 (7.3)	1,546 (16.0)	<0.01	2,688 (14.7)	1,350 (14.7)	0.89
Hyperthyroidism/thyroiditis	1,468 (0.8)	475 (4.9)	<0.01	734 (4.0)	340 (3.7)	0.24
Pancreatitis	163 (0.1)	44 (0.5)	<0.01	55 (0.3)	37 (0.4)	0.16
Bronchiectasis	3,316 (1.7)	431 (4.5)	<0.01	728 (4.0)	373 (4.1)	0.70
Hepatitis C virus infection	1,083 (0.6)	220 (2.3)	<0.01	337 (1.8)	183 (2.0)	0.36
Non-tuberculous mycobacteria infection	106 (0.05)	13 (0.13)	<0.01	26 (0.1)	10 (0.1)	0.48
*Helicobacter pylori* infection	1,248 (0.6)	157 (1.6)	<0.01	290 (1.6)	135 (1.5)	0.49
Ankylosing spondylitis	90 (0.1)	117 (1.2)	<0.01	30 (0.2)	27 (0.3)	0.02
Osteoporosis	4,370 (2.3)	823 (8.5)	<0.01	1,370 (7.5)	702 (7.7)	0.58

*SS, Sjögren's syndrome; PSM, propensity score-matching*.

a *Frequency of outpatient department visits within 1 year of diagnosis with SS*.

### Association of the Risk of Primary Sjögren's Syndrome With Mastitis and Fibrocystic Breast Disease

A conditional logistic regression model adjusted for common medical disorders found that, compared with non-SS controls, SS cases were more likely to have a history of fibrocystic breast disease [aOR, 1.75; 95% confidence interval (CI) 1.63–1.88], cardiovascular diseases (coronary artery disease: aOR, 1.92 and 95% CI, 1.77–2.08; cerebral vascular accident: aOR, 1.63 and 95% CI, 1.47–1.81; hyperlipidaemia: aOR, 2.23 and 95% CI, 2.08–2.38), thyroid diseases (aOR, 5.06 and 95% CI, 4.53–5.66), pancreatitis (aOR, 3.93; 95% CI, 2.75–5.62), bronchiectasis (aOR, 2.22; 95% CI, 1.99–2.47), infectious diseases (hepatitis C virus infection: aOR, 3.25 and 95% CI, 2.78–3.79; *H. pylori* infection: aOR, 1.87 and 95% CI, 1.57–2.23), osteoporosis (aOR, 3.35; 95% CI, 3.07–3.64), and ankylosing spondylitis (aOR, 17.17; 95% CI, 12.82–23.01) (Table 2). Patients with history of mastitis also tended to be associated with incident SS although not reach statistical significance due to a relatively small number of patients with mastitis. In the propensity-score-matched subjects, the association between fibrocystic breast disease and incident SS remained robust using the conditional logistical regression model (aOR, 1.74 and 95% CI, 1.58–1.91) (Table 2).

**Table 2:** Conditional logistical regressions for the estimation of the risk for SS.

	**1:20 age-matched population**	**1:2 PSM population**
	**aOR, 95%CI**	**aOR, 95%CI**
**Fibrocystic disease**	1.75 (1.63–1.88)	1.74 (1.58–1.91)
**Mastitis**	1.08 (0.94–1.23)	1.13 (0.95–1.33)
**Urbanization**		
Urban	Ref.	
Suburban	1.03 (0.98–1.08)	
Rural	1.01 (0.95–1.07)	
**Low income**	0.90 (0.87–0.95)	
**High frequency of outpatient visit^a^**	2.89 (2.75–3.04)	
**Common medical disorders**	
Diabetes mellitus, type I	1.47 (0.89–2.41)	
Diabetes mellitus, type II	0.61 (0.56–0.66)	
Coronary artery disease	1.92 (1.77–2.08)	
Cerebral vascular accident	1.63 (1.47–1.81)	
Hyperlipidaemia	2.23 (2.08–2.38)	
Hyperthyroidism/thyroiditis	5.06 (4.53–5.66)	
Pancreatitis	3.93 (2.75–5.62)	
Bronchiectasis	2.22 (1.99–2.47)	
Hepatitis C virus infection	3.25 (2.78–3.79)	
Non-tuberculous mycobacteria infection	1.55 (0.85–2.83)	
*Helicobacter pylori* infection	1.87 (1.57–2.23)	
Ankylosing spondylitis	17.17 (12.82–23.01)	
Osteoporosis	3.35 (3.07–3.64)	

*SS, Sjögren's syndrome; PSM, propensity score-matching*.

a *Frequency of outpatient department visits within 1 year of diagnosis with pSS higher than median frequency (14.5)*.

### Sensitivity and Subgroup Analyses

The sensitivity analyses revealed that the correlation between pSS and mastitis and fibrocystic breast disease remained consistent using various definitions based on distinct outpatient visit frequencies for mastitis/fibrocystic breast disease (Table 3). In subgroup analyses, the association between the risk of SS and a history of fibrocystic breast disease appeared to be prominent among those older than 40 years (40–65 group, aOR 1.75, 95% CI 1.57–1.95; >65 group, aOR 2.23, 95% CI 1.69–2.93) in propsensity score matched subjects. Similar trend was found in those with history of mastitis (40–65 group, aOR 1.26, 95% CI 1.01–1.57; >65 group, aOR 1.59, 95% CI 0.93–2.73) (Table 4). Consistent results were found in 1:20 matched subjects (see Supplementary Table 1). Collectively, we discovered the association between benign breast diseases, including mastitis and fibrocystic breast disease, and the development of pSS.

**Table 3:** Sensitivity analysis in the estimation of the SS risk for exposure in the age-matched population.

**Distinct definitions**	aOR^*^ (95%CI)
**Fibrocystic disease**	
At least one outpatient visit or one admission (main finding)	1.79 (1.67–1.92)
At least two outpatient visits or 1 admission	1.81 (1.64–1.99)
At least three outpatient visits or 1 admission	1.75 (1.55–1.98)
**Mastitis**	
At least one outpatient visit or one admission (main finding)	1.08 (0.94–1.23)
At least two outpatient visits or one admission	1.05 (0.83–1.32)
At least three outpatient visits or one admission	1.13 (0.83–1.53)

* *Adjusted odds ratio of benign breast disease for the risk of SS, the covariates including age group, urbanization, low income, and comorbidities listed in Table 1. SS, Sjögren's syndrome; aOR, adjusted odds ratio; CI, confidence interval*.

**Table 4:** Stratified analyses for the association between benign breast diseases and Sjogren's syndrome risk (1:2 propensity score matched subjects).

	**Fibrocystic breast disease**		**Mastitis**	
	**aOR (95% CI)**	p^*^	**aOR (95% CI)**	p^*^
**Age**		0.012		0.141
≤ 40	1.29 (0.99–1.66)		0.97 (0.71–1.31)	
40–65	1.75 (1.57–1.95)		1.26 (1.01–1.57)	
≥65	2.23 (1.69–2.93)		1.59 (0.93–2.73)	
**Frequency of OPD visit^a^**		0.748		0.439
Lower than median	1.68 (1.38–2.06)		1.05 (0.72–1.55)	
Higher than median	1.73 (1.56–1.93)		1.22 (1.01–1.47)	
**Diabetes mellitus**		0.028		0.608
No	1.64 (1.47–1.82)		1.17 (0.97–1.40)	
Yes	2.11 (1.71–2.60)		1.30 (0.86–1.96)	
**Coronary artery disease**		0.026		0.229
No	1.68 (1.52–1.85)		1.16 (0.97–1.38)	
Yes	2.47 (1.75–3.49)		1.62 (0.84–3.11)	
**Cerebral vascular accident**		0.583		0.700
No	1.71 (1.55–1.89)		1.18 (0.99–1.40)	
Yes	1.82 (1.29–2.57)		1.36 (0.70–2.66)	
**Hyperlipidaemia**		0.017		0.506
No	1.70 (1.54–1.86)		1.18 (0.99–1.40)	
Yes	3.04 (1.68–5.51)		1.47 (0.59–3.70)	
**Hyperthyroidism/Thyroiditis**		0.610		0.100
No	1.70 (1.53–1.88)		1.12 (0.93–1.34)	
Yes	1.84 (1.47–2.30)		1.66 (1.08–2.54)	
**Bronchiectasis**		0.874		0.123
No	1.72 (1.56–1.89)		1.22 (1.03–1.45)	
Yes	1.78 (1.14–2.75)		0.79 (0.39–1.61)	
**Hepatitis C**		0.908		NA
No	1.72 (1.56–1.89)		1.18 (1.001–1.40)	
Yes	>999 (–)		NA	
***H. pylori*** **infection**		0.090		0.599
No	1.75 (1.59–1.93)		1.19 (1.003–1.41)	
Yes	1.26 (0.81–1.98)		0.95 (0.34–2.62)	
**NTM infection**		0.661		0.526
No	1.72 (1.56–1.89)		1.18 (0.99–1.39)	
Yes	1.95 (0.99–3.82)		1.96 (0.65–5.93)	
**Ankylosing spondylitis**		0.946		NA
No	1.72 (1.57–1.89)		1.19 (1.002–1.40)	
Yes	1.69 (0.05–59.91)		NA	
**Osteoporosis**		0.502		0.466
No	1.73 (1.57–1.90)		1.20 (1.01–1.42)	
Yes	1.58 (0.88–2.81)		0.79 (0.24–2.57)	

*OPD, outpatient department; NTM, nontuberculous mycobacteria*.

* *p for interaction*.

a *Frequency of outpatient department visits within 1 year of diagnosis of pSS*.

## Discussion

Primary Sjögren's syndrome affects the exocrine glands, including the salivary, lacrimal, and mammary glands; however, few studies explored mammary-gland-inflammation-associated breast diseases compared with numerous lines of evidence regarding xerostomia and xerophthalmia in patients with pSS. In this population-based study, we found that fibrocystic breast disease was consistently associated with pSS in both age-matched and propensity-score-matched populations. Our findings indicate the need for collaboration between gynecologists and rheumatologists to survey pSS among middle-aged female patients with benign breast diseases, particularly fibrocystic disease.

The pathogenesis of SS can be attributed to genetic susceptibility and an epigenetic effect of hormone levels and environmental factors, and clinical manifestations of SS consist of gradual development of autoantibody as well as symptoms of exocrine system, with exacerbation due to environmental triggers (16). We postulated that the preceding fibrocystic breast diseases in patients with SS may result from not only from a diagnostic delay due to subtle early signs of inflammation in the other exocrine glands, but also from possible shared immunological mechanisms between fibrocystic breast disease and SS. In line with our finding that the interval between fibrocystic breast disease manifestation and the diagnosis of SS was approximately 5 years (Table 1), previous studies have estimated that the median delay between the onset of the symptoms of these diseases and the diagnosis of pSS was 2–6 years (17). Unlike the numerous studies that investigated the association between xeropthalmia/xerostomia and pSS (3, 4), evidence regarding the association between mammary-gland-inflammation–associated diseases and SS is apparently scarce (18). Therefore, there are opportunities for, and challenges in the recognition of subtle symptoms of pSS for the early diagnosis of this condition in patients with the benign breast disease. These lines of evidence indicate the urgent need for collaboration with rheumatologists to survey in detail pSS in patients with fibrocystic diseases, particularly in middle-aged women.

Exocrine glandular inflammation with peri-duct lymphocytic cells that may merged in focus is one of the hallmark histological findings of labial salivary biopsy among patients with SS (19). Notably, the aforementioned findings in salivary gland share anatomical, histological, and immunological features with mammary glands as demonstrated by Goulabchand et al. through examining histology of the salivary gland and mammary tissues among nine SS patients with benign breast diseases (20). In fact, the aberrant inflammation that occurs in the pathogenesis of SS consists of complex immune responses, including deregulated inflammation against environmental microorganisms (21, 22). Furthermore, previous studies, including our previous epidemiological study, have found that non-tuberculous infection was associated with pSS, possibly through a shared type I interferon response (12, 23). Katsifis et al. identified increased levels of interleukin-17 in the plasma and exocrine glandular tissues of patients with pSS (24); in addition, early increased production of IL-17 in the mammary glands was also found to improve the outcome of mastitis (25). Similarly, the signal transduction and activator of transcription 3 (STAT3) and the nuclear factor-κB (NFκB) signaling pathways have been implicated in mastitis (26), and were also found to be crucial signaling pathways in the pathogenesis of pSS (27). Therefore, the association between benign breast disease and SS, as demonstrated in the present study, suggests the existence of a shared immunological pathway between these two inflammatory diseases.

In the present study, we found a strong association between AS and pSS. Several studies, mainly case reports and case series, have also reported an association between AS and pSS (28–30). Kobak et al. conducted a single-center study to evaluate the frequency of pSS in 70 patients with AS in 2002–2003 and found that 10% (7/70) of the patients with AS had concomitant pathologically proven pSS (29). Recently, Usuba et al. examined the condition of eyes in 36 consecutive patients with AS and reported that 38.8% (14/36) of these patients had an abnormal Schirmer test (<10 mm/5 min) (30). Notably, Usuba et al. further investigated the dry eye condition among 14 patients with AS who underwent tumor necrosis factor inhibitor (TNFi) therapy at 3 and 12 months after the administration of TNFi, and found that Schirmer's test at the baseline, 3 and 12 months was 10, 17.5, and 20 mm/5 min, respectively (30). The aforementioned study highlights the largely ignored presence of xerophthalmia in patients with AS and the therapeutic potential of TNFi to alleviate this condition. However, a large-scale study addressing SS in patients with AS is still lacking. In this population-based study, we identified a robust association between AS and SS, which indicates an essential need to survey xerophthalmia and potential pSS in patients with AS.

We also noted that osteoporosis appeared to be associated with a modestly increased risk of developing pSS, and the finding was consistent with previous studies (1, 31, 32). One Taiwanese study reported that the presence of osteoporosis was associated with a slightly increased risk of developing xerophthalmia (aOR, 1.26; 95% CI, 1.22–1.30) (31). In fact, pSS is apparently prevalent in post-menopausal women, with the female/male ratio of SS being 10.72 (95% CI, 7.35–15.62) and the overall age being 56.16 (95% CI, 52.54–59.78) years (1). The strong predisposition of post-menopausal women to develop pSS suggests a key role for sex hormones in the pathogenesis of pSS. Notably, McCoy et al. recently conducted a case–control study of 2,680 women in the Sjögren's International Collaborative Clinical Alliance registry and found that pSS in women was associated with a lower cumulative estrogen exposure and the menstrual cycling time compared with sicca controls (32). The aforementioned evidence and the central role of estrogen deficiency in the pathogenesis of osteoporosis suggest that a low estrogen level contributes to the association between osteoporosis and SS in post-menopausal women.

The large number of patients with pSS included in the present study enabled us to validate the associations between SS and cardiovascular diseases, infectious diseases and glandular-epithelial-inflammation–related diseases. Several studies have shown that pSS is associated with incident cardiovascular diseases (33–35). In the present study, we further found that patients with cardiovascular diseases (including coronary artery disease, hyperlipidaemia, and cerebral vascular accident) had a slightly increased risk of developing pSS. The aforementioned association between pSS and cardiovascular disease might result from the co-existence of the two diseases with a distinct time of diagnosis. In line with the previous study, we found that type I diabetes mellitus tended to be associated with incident pSS, although statistical significance was not reached (36). Intriguingly, type II diabetes mellitus exhibited an inverse association with incident SS in multivariable regression; thus, we speculated that hyperglycaemia might potentially alter the immunogenicity of autoimmunity, similar to the hyperglycaemia-associated increase in the number of splenic regulatory T cells in the autoimmune pancreatitis mouse model (37). In line with the findings of the present study, previous studies, including our recently published work, have shown that infectious diseases and medications for infectious diseases, including hepatitis C virus infection and *H. pylori* infection, tended to be associated with incident SS (12, 38). In addition, epithelial cells, which are the target cells in pSS, drive the pathogenesis of SS by promoting an aberrant immune response (16, 39), and the glandular epithelium lymphoplasmacytic infiltrate in the bronchial wall, pancreas, thyroid gland, and mammary gland may be responsible for the strong association detected among these glandular inflammation diseases, as shown here.

There were several limitations in this study that merit discussion. First, the accuracy of the diagnosis based on the ICD code is of concern. However, the accuracy of the pSS diagnosis is less problematic because it was validated by two qualified rheumatologists in the process of issuing a catastrophic illness certificate. Second, potential unmeasured confounders, such as smoking, environmental exposure and lifestyle-relevant variables, cannot be assessed in NHIRD; however, the adjustment for socioeconomic status performed in the present study should mitigate this issue, at least partly. Moreover, the lack of serological and clinical data in claim data is a limitation. Third, a causal inference would be imprudent in this epidemiological study; however, the present population-based study provides evidence of a previously unreported association between mammary-gland-inflammation–associated diseases and SS. Fourth, the association between mastitis and pSS cannot be delineated due to a relatively lower number of subjects with mastitis compared those with fibrocystic breast disease.

## Conclusions

Exocrine glandular inflammation is the hallmark pathogenesis in patients with SS; however, studies focusing on the association between mammary-gland-inflammation–relevant diseases and pSS are lacking. The present population-based case–control study identified a previously unexplored association between fibrocystic breast disease and pSS, and validated several risk factors for this disease. These findings indicate an essential need for collaboration with rheumatologists to survey pSS during the management of patients with fibrocystic breast disease. Finally, additional studies are required to elucidate the mechanisms underlying these associations.

## Data Availability Statement

The original contributions presented in the study are included in the article/Supplementary Material, further inquiries can be directed to the corresponding author/s.

## Ethics Statement

The studies involving human participants were reviewed and approved by Taichung Veterans General Hospital (CE19038A). The ethics committee waived the requirement of written informed consent for participation.

## Author Contributions

Conceived and designed the experiments: H-HC, K-TT, D-YC, JW, and W-CC. Acquired data: H-HC, C-HL, and W-CC. Contributed materials/analysis tools: H-HC, H-MC, and W-CC. Wrote the paper: H-HC, JW, and W-CC. All authors contributed to the article and approved the submitted version.

## Conflict of Interest

The authors declare that the research was conducted in the absence of any commercial or financial relationships that could be construed as a potential conflict of interest.

## References

[1] Qin B Wang J Yang Z Yang M Ma N Huang F. Epidemiology of primary Sjogren's syndrome: a systematic review and meta-analysis. Ann Rheum Dis. (2015) 74:1983–9. 10.1136/annrheumdis-2014-205375 24938285

[2] Mavragani CP Moutsopoulos HM. The geoepidemiology of Sjogren's syndrome. Autoimmun Rev. (2010) 9:A305–10. 10.1016/j.autrev.2009.11.004 19903539

[3] Sharma R Chaudhari KS Kurien BT Grundahl K Radfar L Lewis DM. Sjogren syndrome without focal lymphocytic infiltration of the salivary glands. J Rheumatol. (2020) 47:394–99. 10.3899/jrheum.181443 31092717 PMC7304293

[4] Parkin B Chew JB White VA Garcia-Briones G Chhanabhai M Rootman J. Lymphocytic infiltration and enlargement of the lacrimal glands: a new subtype of primary Sjogren's syndrome? Ophthalmology. (2005) 112:2040–7. 10.1016/j.ophtha.2005.06.014 16168486

[5] Fox PC. Autoimmune diseases and Sjogren's syndrome: an autoimmune exocrinopathy. Ann N Y Acad Sci. (2007) 1098:15–21. 10.1196/annals.1384.003 17332090

[6] Goulabchand R Hafidi A Van de Perre P Millet I Maria ATJ Morel J. Mastitis in autoimmune diseases: review of the literature, diagnostic pathway, and pathophysiological key players. J Clin Med. (2020) 9:958. 10.3390/jcm9040958 32235676 PMC7231219

[7] Santen RJ Mansel R. Benign breast disorders. N Engl J Med. (2005) 353:275–85. 10.1056/NEJMra035692 16034013

[8] Saboo A Bennett I. Trends in non-lactation breast abscesses in a tertiary hospital setting. ANZ J Surg. (2018) 88:739–44. 10.1111/ans.14146 29045009

[9] Berens PD. Breast pain: engorgement, nipple pain, and mastitis. Clin Obstet Gynecol. (2015) 58:902–14. 10.1097/GRF.0000000000000153 26512442

[10] Ader DN Browne MW. Prevalence and impact of cyclic mastalgia in a United States clinic-based sample. Am J Obstet Gynecol. (1997) 177:126–32. 10.1016/s0002-9378(97)70450-2 9240595

[11] Fitzstevens JL Smith KC Hagadorn JI Caimano MJ Matson AP Brownell EA. Systematic review of the human milk microbiota. Nutr Clin Pract. (2017) 32:354–64. 10.1177/0884533616670150 27679525

[12] Chao WC Lin CH Liao TL Chen YM Chen DY Chen HH. Association between a history of mycobacterial infection and the risk of newly diagnosed Sjogren's syndrome: a nationwide, population-based case-control study. PLoS ONE. (2017) 12:e0176549. 10.1371/journal.pone.0176549 28486537 PMC5423582

[13] Chao WC Lin CH Chen YM Hsu CY Chen JP Chen HH. Associations between Antibiotics for non-tuberculous Mycobacterial infection and incident Sjogren's syndrome: a nationwide, population-based case-control study. Sci Rep. (2018) 8:16007. 10.1038/s41598-018-34495-4 30375488 PMC6207743

[14] Lin YJ Tian WH Chen CC. Urbanization and the utilization of outpatient services under National Health Insurance in Taiwan. Health Policy. (2011) 103:236–43. 10.1016/j.healthpol.2011.08.007 21920621

[15] Lu MC Fa WH Tsai TY Koo M Lai NS. Increased utilisation of eye disorder-related ambulatory medical services prior to the diagnosis of Sjogren's syndrome in female patients: a longitudinal population-based study in Taiwan. BMJ Open. (2014) 4:e003862. 10.1136/bmjopen-2013-003862 24844268 PMC4039788

[16] Nocturne G Mariette X. Advances in understanding the pathogenesis of primary Sjogren's syndrome. Nat Rev Rheumatol. (2013) 9:544–56. 10.1038/nrrheum.2013.110 23857130

[17] Wang B Chen S Zheng Q Li Y Zhang X Xuan J. Early diagnosis and treatment for Sjogren's syndrome: current challenges, redefined disease stages and future prospects. J. Autoimmun. (2020) 117:102590. 10.1016/j.jaut.2020.102590 33310686

[18] Rios G Peredo RA. Lymphocytic mastitis preceding Sjogren's syndrome. P R Health Sci J. (2010) 29:127–9. 20496529

[19] Fisher BA Jonsson R Daniels T Bombardieri M Brown RM Morgan P. Standardisation of labial salivary gland histopathology in clinical trials in primary Sjogren's syndrome. Ann Rheum Dis. (2017) 76:1161–68. 10.1136/annrheumdis-2016-210448 27965259 PMC5530351

[20] Goulabchand R Hafidi A Millet I Morel J Lukas C Humbert S. Mastitis associated with Sjogren's syndrome: a series of nine cases. Immunol Res. (2017) 65:218–29. 10.1007/s12026-016-8830-x 27561784

[21] Pontarini E Lucchesi D Bombardieri M. Current views on the pathogenesis of Sjogren's syndrome. Curr Opin Rheumatol. (2018) 30:215–21. 10.1097/BOR.0000000000000473 29227354

[22] Imgenberg-Kreuz J Rasmussen A Sivils K Nordmark G. Genetics and epigenetics in primary Sjogren's syndrome. Rheumatology (Oxford). (2021) 60:2085–98. 10.1093/rheumatology/key330 30770922 PMC8121440

[23] Clayton K Polak ME Woelk CH Elkington P. Gene Expression signatures in tuberculosis have greater overlap with autoimmune diseases than with infectious diseases. Am J Respir Crit Care Med. (2017) 196:655–56. 10.1164/rccm.201706-1248LE 28753379 PMC5620671

[24] Katsifis GE Rekka S Moutsopoulos NM Pillemer S Wahl SM. Systemic and local interleukin-17 and linked cytokines associated with Sjogren's syndrome immunopathogenesis. Am J Pathol. (2009) 175:1167–77. 10.2353/ajpath.2009.090319 19700754 PMC2731135

[25] Porcherie A Gilbert FB Germon P Cunha P Trotereau A Rossignol C. IL-17A is an important effector of the immune response of the mammary gland to *Escherichia coli* infection. J Immunol. (2016) 196:803–12. 10.4049/jimmunol.1500705 26685206

[26] Sargeant TJ Lloyd-Lewis B Resemann HK Ramos-Montoya A Skepper J Watson CJ. Stat3 controls cell death during mammary gland involution by regulating uptake of milk fat globules and lysosomal membrane permeabilization. Nat Cell Biol. (2014) 16:1057–68. 10.1038/ncb3043 25283994 PMC4216597

[27] Sisto M Ribatti D Lisi S. Understanding the complexity of Sjogren's syndrome: remarkable progress in elucidating NF-kappaB mechanisms. J Clin Med. (2020) 9:2821. 10.3390/jcm9092821 32878252 PMC7563658

[28] Zhao GW Huang LF Li D Zeng Y. Ankylosing spondylitis coexists with rheumatoid arthritis and Sjogren's syndrome: a case report with literature review. Clin Rheumatol. (2020) 40:2083–6. 10.1007/s10067-020-05350-7 32936426

[29] Kobak S Kobak AC Kabasakal Y Doganavsargil E. Sjogren's syndrome in patients with ankylosing spondylitis. Clin Rheumatol. (2007) 26:173–5. 10.1007/s10067-006-0255-9 16547690

[30] Usuba FS Saad CGS Aikawa NE Novaes P Moraes JCB Santo RM. Improvement of conjunctival cytological grade and tear production in ankylosing spondylitis patients under TNF inhibitors: a long-term follow-up. Sci Rep. (2020) 10:334. 10.1038/s41598-019-57266-1 31942038 PMC6962203

[31] Jeng YT Lin SY Hu HY Lee OK Kuo LL. Osteoporosis and dry eye syndrome: a previously unappreciated association that may alert active prevention of fall. PLoS ONE. (2018) 13:e0207008. 10.1371/journal.pone.0207008 30395639 PMC6218084

[32] McCoy SS Sampene E Baer AN. Association of Sjogren's syndrome with reduced lifetime sex hormone exposure: a case-control study. Arthritis Care Res (Hoboken). (2020) 72:1315–22. 10.1002/acr.24014 31233285 PMC6928446

[33] Bartoloni E Baldini C Schillaci G Quartuccio L Priori R Carubbi F. Cardiovascular disease risk burden in primary Sjogren's syndrome: results of a population-based multicentre cohort study. J Intern Med. (2015) 278:185–92. 10.1111/joim.12346 25582881

[34] Yong WC Sanguankeo A Upala S. Association between primary Sjogren's syndrome, cardiovascular and cerebrovascular disease: a systematic review and meta-analysis. Clin Exp Rheumatol. (2018) 36(Suppl 112):190–97. 29600936

[35] Beltai A Barnetche T Daien C Lukas C Gaujoux-Viala C Combe B. Cardiovascular morbidity and mortality in primary sjogren's syndrome: a systematic review and meta-analysis. Arthritis Care Res (Hoboken). (2020) 72:131–39. 10.1002/acr.23821 30570824

[36] Bao YK Weide LG Ganesan VC Jakhar I McGill JB Sahil S. High prevalence of comorbid autoimmune diseases in adults with type 1 diabetes from the HealthFacts database. J Diabetes. (2019) 11:273–79. 10.1111/1753-0407.12856 30226016

[37] Muller-Graff FT Fitzner B Jaster R Vollmar B Zechner D. Impact of hyperglycemia on autoimmune pancreatitis and regulatory T-cells. World J Gastroenterol. (2018) 24:3120–29. 10.3748/wjg.v24.i28.3120 30065558 PMC6064968

[38] Argyropoulou OD Pezoulas V Chatzis L Critselis E Gandolfo S Ferro F. Cryoglobulinemic vasculitis in primary Sjogren's Syndrome: clinical presentation, association with lymphoma and comparison with Hepatitis C-related disease. Semin Arthritis Rheum. (2020) 50:846–53. 10.1016/j.semarthrit.2020.07.013 32896698

[39] Gong YZ Nititham J Taylor K Miceli-Richard C Sordet C Wachsmann D. Differentiation of follicular helper T cells by salivary gland epithelial cells in primary Sjogren's syndrome. J.Autoimmun. (2014) 51:57–66. 10.1016/j.jaut.2013.11.003 24411167

