# Supplementary material for: Association of the Risk of Primary Sjögren's Syndrome With Fibrocystic Breast Disease: A Nationwide, Population-Based Study
Source: Front Med (Lausanne). 2021 Jul 1;8:704593. doi: 10.3389/fmed.2021.704593 (PMC8280500; doi:10.3389/fmed.2021.704593)
Supplement: Supplementary file 1 [file Table_1.docx]

| **Supplemental table 1. Stratified analyses for the association between benign breast diseases and Sjogren's syndrome risk (1:20 matched subjects)** | | | | |
| --- | --- | --- | --- | --- |
|  | **Fibrocystic breast disease** | | **Mastitis** | |
|  | aOR (95% CI) | p* | aOR (95% CI) | p* |
| **Age** |  | 0.026 |  | 0.026 |
| ≦40 | 1.43 (1.17–1.74) |  | 1.43 (1.17–1.74) |  |
| 40-65 | 1.81 (1.67–1.97) |  | 1.81 (1.67–1.97) |  |
| ≧65 | 3.19 (3.17–3.20) |  | 3.19 (3.17–3.20) |  |
| **Frequency of OPD visit^a^** |  | 0.636 |  | 0.636 |
| Lower than median | 1.73 (1.47–2.05) |  | 1.73 (1.47–2.05) |  |
| Higher than median | 1.80 (1.67–1.95) |  | 1.80 (1.67–1.95) |  |
| **Diabetes mellitus** |  | 0.091 |  | 0.091 |
| No | 1.71 (1.70–1.71) |  | 1.71 (1.70–1.71) |  |
| Yes | 2.32 (2.31–2.33) |  | 2.32 (2.31–2.33) |  |
| **Coronary artery disease** |  | 0.001 |  | 0.001 |
| No | 1.74 (1.61–1.87) |  | 1.74 (1.61–1.87) |  |
| Yes | 2.69 (2.10–3.45) |  | 2.69 (2.10–3.45) |  |
| **Cerebral vascular accident** |  | 0.96 |  | 0.96 |
| No | 1.79 (1.67–1.93) |  | 1.79 (1.67–1.93) |  |
| Yes | 1.73 (1.33–2.25) |  | 1.73 (1.33–2.25) |  |
| **Hyperlipidaemia** |  | 0.018 |  | 0.018 |
| No | 1.76 (1.76–1.76) |  | 1.76 (1.76–1.76) |  |
| Yes | 2.50 (1.67–3.74) |  | 2.50 (1.67–3.74) |  |
| **Hyperthyroidism/Thyroiditis** |  | 0.014 |  | 0.014 |
| No | 1.74 (1.61–1.88) |  | 1.74 (1.61–1.88) |  |
| Yes | 2.07 (1.74–2.45) |  | 2.07 (1.74–2.45) |  |
| **Bronchiectasis** |  | 0.736 |  | 0.736 |
| No | 1.79 (1.66–1.92) |  | 1.79 (1.66–1.92) |  |
| Yes | 1.85 (1.29–2.65) |  | 1.85 (1.29–2.65) |  |
| **Hepatitis C** |  | 0.652 |  | 0.652 |
| No | 1.79 (1.67–1.92) |  | 1.79 (1.67–1.92) |  |
| Yes | 1.83 (0.40–8.32) |  | 1.83 (0.40–8.32) |  |
| ***H. pylori* infection** |  | 0.174 |  | 0.174 |
| No | 1.81 (1.68–1.95) |  | 1.81 (1.68–1.95) |  |
| Yes | 1.46 (1.01–2.10) |  | 1.46 (1.01–2.10) |  |
| **NTM infection** |  | 0.778 |  | 0.778 |
| No | 1.78 (1.78–1.79) |  | 1.78 (1.78–1.79) |  |
| Yes | 1.77 (1.03–3.04) |  | 1.77 (1.03–3.04) |  |
| **Ankylosing spondylitis** |  | 0.992 |  | 0.992 |
| No | 1.79 (1.67–1.93) |  | 1.79 (1.67–1.93) |  |
| Yes | 1.54 (0.20–12.03) |  | 1.54 (0.20–12.03) |  |
| **Osteoporosis** |  | 0.951 |  | 0.951 |
| No | 1.79 (1.67–1.92) |  | 1.79 (1.67–1.92) |  |
| Yes | 1.98 (1.24–3.14) |  | 1.98 (1.24–3.14) |  |
| *p for interaction. ^a^Frequency of outpatient department visits within one year of diagnosis of pSS. Abbreviations: OPD, outpatient department; NTM, nontuberculous mycobacteria. | | | | |
|  |  |  |  |  |
